# The Associations between Regional Gray Matter Structural Changes and Changes of Cognitive Performance in Control Groups of Intervention Studies

**DOI:** 10.3389/fnhum.2015.00681

**Published:** 2015-12-21

**Authors:** Hikaru Takeuchi, Yasuyuki Taki, Yuko Sassa, Atsushi Sekiguchi, Tomomi Nagase, Rui Nouchi, Ai Fukushima, Ryuta Kawashima

**Affiliations:** ^1^Smart Ageing International Research Center, Institute of Development, Aging and Cancer, Tohoku UniversitySendai, Japan; ^2^Division of Developmental Cognitive Neuroscience, Institute of Development, Aging and Cancer, Tohoku UniversitySendai, Japan; ^3^Department of Functional Brain Imaging, Institute of Development, Aging and Cancer, Tohoku UniversitySendai, Japan; ^4^Faculty of Medicine, Tohoku UniversitySendai, Japan

**Keywords:** cognitive tests, learning, plasticity, voxel based morphometry, dorsal anterior cingulate gyrus, superior frontal gyrus

## Abstract

In intervention studies of cognitive training, the challenging cognitive tests, which were used as outcome measures, are generally completed in more than a few hours. Here, utilizing the control groups' data from three 1-week intervention studies in which young healthy adult subjects underwent a wide range of cognitive tests and T1-weighted magnetic resonance imaging (MRI) before and after the intervention period, we investigated how regional gray matter (GM) density (rGMD) of the subjects changed through voxel-based morphometry (VBM). Statistically significant increases in rGMD were observed in the anatomical cluster that mainly spread around the bilateral dorsal anterior cingulate cortex (dACC) and the right superior frontal gyrus (rSFG). Moreover, mean rGMD within this cluster changes were significantly and positively correlated with performance changes in the Stroop task, and tended to positively correlate with performance changes in a divergent thinking task. Affected regions are considered to be associated with performance monitoring (dACC) and manipulation of the maintained information including generating associations (rSFG), and both are relevant to the cognitive functions measured in the cognitive tests. Thus, the results suggest that even in the groups of the typical “control group” in intervention studies including those of the passive one, experimental or non-experimental factors can result in an increase in the regional GM structure and form the association between such neural changes and improvements related to these cognitive tests. These results suggest caution toward the experimental study designs without control groups.

## Introduction

Since it has been recognized that the regional gray matter (GM) structure is affected by cognitive training (Draganski et al., 2006), numerous studies have reported that brain structure is affected by a wide range of cognitive interventions (Boyke et al., 2008; Driemeyer et al., 2008; Ilg et al., 2008; Takeuchi et al., 2011c,d). Growing evidence indicates that GM structure can be easily changed by just a 1-week cognitive intervention (Driemeyer et al., 2008; Quallo et al., 2009; Takeuchi et al., 2011c,d).

On the contrary, it is well known that the first exposure to a cognitive test substantially improves performance on the outcome measures in the second test when the tests do not involve knowledge (Wechsler, 1997).

We previously proposed that exposure to cognitive tests that are used as outcome measures in intervention studies may lead to changes in GM structure (Takeuchi et al., 2011d). In intervention studies of cognitive training, in addition to MRI scans, a wide range of cognitive tests is often used as outcome measures, and trend level improvements are observed in performance on these outcome measures in the second test (Olesen et al., 2004; McNab and Klingberg, 2007; Dahlin et al., 2008; Takeuchi et al., 2011d). In our previous studies, tests used as outcome measures were usually completed within 3–4 h. Considering that in intervention studies of cognitive training, training typically lasts for 10–20 h (Takeuchi et al., 2010c), 3–4 h of training is not negligible. These tests were not performed using the adaptive procedures known to improve cognitive functions in working memory training (Klingberg et al., 2002); non-adaptive low-level training does not cause any improvement in cognitive functions nor plasticity of brain structure (Klingberg et al., 2002; Takeuchi et al., 2011c). However, in most cases, (a) these tests are performed progressively, i.e., problems become increasingly difficult, or (b) problems have to be solved rapidly, i.e., the participants are asked to solve as many problems as quickly as possible in a given time. Thus, these tests challenge the cognitive limits of the subjects, unlike low level non-adaptive training used in such studies. Therefore, groups that do not receive the experimental intervention but undergo these cognitive tests may display intervention-irrelevant changes in brain structure. However, thus far, this is simply a hypothesis, and whether this hypothesis is true and whether control groups that underwent cognitive tests in the intervention studies display these structural changes are not known.

We hypothesized that participation as passive and non-adaptive low-level active control groups, which were exposed to 1-day cognitive tests, resulted in increases in regional GM a week later in young healthy adults and (b) that the increases are positively associated with learning how to perform outcome measures (or pre- to post-test improvement in performance on outcome measures). Specifically, we expected the regional GM would increase from the pre-scan to the post-scan. This is consistent with our proposition that intensive adaptive training would cause non-linear regional GM changes (first increase then decrease; note here in this study, the expected change corresponds to the first increase; Takeuchi et al., 2011c) as was the case with developmental regional GM change (Sowell et al., 2003). Regarding the candidate regions, exposure to these cognitive tests may affect the structure of the network, which mainly consists of the lateral prefrontal cortex, parts of the lateral parietal cortex, and the dorsal anterior cingulate cortex (dACC) (Fox et al., 2005). This is because while subjects are exposed to externally directed attention-demanding cognitive tasks, the areas in this network are consistently activated (Fox et al., 2005). Although all of these areas in this network or the network itself is considered important for a wide range of cognitive tasks (Jung and Haier, 2007), dACC in particular is functionally and structurally suggested to play a key role in tasks such as the Stroop task (Laird et al., 2005; Takeuchi et al., 2012b), whereas for the cognitive tasks requiring manipulation of maintained information such as divergent thinking and reasoning, the dorsal part of the lateral prefrontal cortex (DLPFC) may be more important (Duncan et al., 2000; Takeuchi et al., 2010b).

The purpose of this study was to test these hypotheses and reveal whether control groups that were exposed to cognitive tests in the intervention studies exhibit structural changes and whether these changes are associated with increased cognitive performance. To confirm these hypotheses, we used data of 4 control groups from three 1-week intervention studies. Three of these control groups constituted passive control groups, while 1 constituted the low level non-adaptive active control group; this low level non-adaptive active control training (for details, see Methods) has no effects on cognitive functions or brain structures (for results, see Takeuchi et al., 2011c). In these studies, before and after intervention, subjects underwent T1-weighted MRI (structural images were obtained) and psychological experiments in which a wide range of cognitive outcome measures were assessed within the same session. Using voxel-based morphometry (VBM), changes in regional gray matter density (rGMD) after intervention as well as associations of these changes with performance changes in outcome measures were calculated. In intervention studies, traditionally, control groups have not always been set, and the effects of the intervention have been tested by demonstrating the association between neural changes and changes in outcome measures in the experimental intervention group. Thus, revealing whether these neural changes and changes in outcome measures occur without the experimental intervention is important.

## Methods

### Subjects

Data from subjects who were randomly assigned to the low-level non-adaptive active control group or passive control groups and had completed the experimental procedures in our 2 previously published studies (Takeuchi et al., 2011c,d) as well as 1 unpublished study were included in this study. The first study included 2 control groups, namely low-level non-adaptive active control training and passive control (passive control group 1) groups. The second and third studies both included 1 passive control group (passive control groups 2 and 3). In each experiment, neither subjects nor the experimenters could choose to what groups they would be assigned, and they were assigned to various groups in a random manner (assignment was performed in a non-arbitrary manner). The details of the procedures used in group assignments are described in our previous studies (Takeuchi et al., 2011c,d), and the group assignments in the unpublished third study were performed in a random manner.

The low-level non-adaptive active control group consisted of 12 men and 6 women (age, 21.6 ± 1.6 years). Passive control group 1 consisted of 17 men and 2 women (age, 21.7 ± 1.3 years). Passive control group 2 consisted of 12 men and 9 women (age, 21.2 ± 1.7 years). Passive control group 3 consisted of 17 men and 11 women (age, 20.4 ± 1.8 years). All subjects of four groups consisted of 58 men and 28 women (age, 21.1 ± 1.7 years).

All subjects were university, college, or post-graduate students or recent graduates (< 1 year) from these institutions, and they had normal vision. We provided our laboratory's routine questionnaire (which were also used and described in our previous studies; e.g., Takeuchi et al., 2013) to all potential experimental subjects for the assessment of psychiatric illnesses and recent drug use history. In the questionnaire, subjects were asked to provide a detailed list of all drugs that they had used recently. None had a history of neurological or psychiatric illness. These assessments, made during recruitment and through questions after recruitment, were based on voluntary self-report. Handedness was evaluated using the Edinburgh Handedness Inventory (Oldfield, 1971). The studies were conducted in accordance with the Declaration of Helsinki (1991). Written informed consent was obtained from each subject. The Ethics Committee of Tohoku University approved the study.

One subject in the low level non-adaptive active control group and 1 in passive control group 2 failed to complete the experimental procedures. Moreover, another 2 subjects misunderstood the rules of the cognitive tasks used as outcome measures (as evidenced by few answers in simple tasks or chance-level accuracy). These 4 subjects were excluded from the study.

### Procedure

The low level non-adaptive control training program consisted of computerized, in-house–developed Borland C++ programs consisting of one mental multiplication task and one mental addition task. Participants in the low level non-adaptive control training group undertook 5 days of training within a 6-day period. In the mental multiplication task, 2-digit times 2-digit multiplication tasks were presented and subjects were asked to perform mental calculation and solve them. In the mental addition task, 10 two-digit numbers were presented individually and the subjects were asked to add them. The difficulty of the tasks did not change from these initial levels in the low-level non-adaptive control training group. For details of the training tasks in the low level non-adaptive control training group, see our previous work (Takeuchi et al., 2011c).

Low level non-adaptive (i.e., the difficulty level was not modulated) training does not cause any improvement in cognitive functions and structural brain changes (Klingberg et al., 2002; Takeuchi et al., 2011c). This training also did not cause any improvement in cognitive functions and structural brain changes when compared to the no-intervention (passive control) group (Takeuchi et al., 2011c). Training on each day lasted for about 4 h. All participants underwent MRI and the psychological tests immediately before and after this 6-day period. Approximately 50% of subjects underwent MRI first, and the remaining subjects underwent the psychological tests first.

The passive control groups 1 and 2 did not receive any training or perform any specific activity during the period separating the 2 MRI sessions and psychological tests. Passive control group 3 completed a session of T1-weighted structural imaging only at night 2 days after the first day of MRI sessions and psychological tests. The total time of psychological tests varied little across different control groups.

### Psychological outcome measures

For pre- and post-training evaluation, a battery of neuropsychological tests and questionnaires was administered in 4 control groups in 3 studies. Different tests were administered for different studies. However, tests were administered within a period of 3–4 h in all experiments. We listed all tests in Table 1 and to which groups each test was administered.

**Table 1:** **Cognitive tests conducted in the experiment**.

	**Test name**	Conducted groups^*^	**Cognitive function**
A	Raven's Advanced Progressive Matrices	A, P1, P2, P3	Non-verbal reasoning
B	The simple arithmetic task	A, P1, P2, P3	Simple arithmetic
C	The complex arithmetic task	A, P1, P2, P3	Complex arithmetic
D	Word-Color task	A, P1, P2, P3	Processing speed
E	Reverse Stroop task	A, P1, P2, P3	Inhibition, executive functions
F	Color-Word task	A, P1, P2, P3	Processing speed
G	Stroop task	A, P1, P2, P3	Inhibition, executive functions
H	The S-A creativity test	A, P1, P2, P3	Divergent thinking
I	The arithmetic task in WAIS-III	A, P1	Verbal working memory
J	Digit symbol substitution	A, P1	Processing speed
K	The letter mental rotation task	A, P1	Mental rotation
L	The letter span task	A, P1	Working memory
M	Trail Making Tests A and B	A, P1	Processing speed (A) Executive function (B)
N	Cattell's Culture Fair Test	P2	Non-verbal reasoning
O	A computerized digit span task	P2, P3	Verbal working memory
P	A computerized visuospatial working memory task	P2, P3	Visuospatial working memory
Q	The Tanaka B-type intelligence test	P2, P3	Intelligence test with speeded tasks
R	A computerized auditory backward operation span	P3	Complex verbal working memory

* *A represents the non-adaptive low-level active control group. P1, P2, and P3 represent passive control groups 1, 2, and 3 respectively*.

In the following tasks, the basic instruction and the practice required by the manuals of tasks, were administered. No feedback of performance from the experimenters was provided.

[A] Raven's Advanced Progressive Matrices (RAPM) (Raven, 1998) is a non-verbal reasoning task. For details on how we used this task, see our previous study (Takeuchi et al., 2010b).
[B, C] Simple and arithmetic tasks are similar to those constructed by Grabner et al. (2007). These tests measure multiplication performance consisting of 2 types of 1-digit times 1-digit multiplication problems (a simple arithmetic task employing numbers between 2 and 9) and 2 types of 2-digit times 2-digit multiplication problems (a complex arithmetic task employing numbers between 11 and 19). The 2 types were similar in each task, but the numbers used in the problems were different. Each type of the simple and complex arithmetic tasks was presented with time limits of 30 and 60 s, respectively.
[D,E,F,G] The Stroop task (Hakoda's version; Hakoda and Sasaki, 1990), which measures response inhibition and impulsivity and which is the matching-type Stroop task. The following description is essentially the same as the description in our previous study (Takeuchi et al., 2012b). Unlike the oral naming-type Stroop tasks, in the matching-type Stroop task (writing), participants had to choose and write down as many appropriate answers as possible from five options. This type of task enables the measurement of participants' performance correctly. The task consists of two control tasks (Word-Color task, Color-Word task), a reverse Stroop task, and a Stroop task. Reverse Stroop interference means the slowing of an output when participants have to provide the meaning of a word when there is a conflict between the meaning of the word and its printed color. In the Word-Color task, a color name (e.g., “blue”) is presented in the leftmost column. In addition, five columns are painted with five different colors and participants have to check the column whose color corresponds to the color name in the leftmost column. In the Color-Word task, the leftmost column is painted with a color, and five other columns contain color names. The participants have to check the column with the word corresponding to the name of the color painted in the leftmost column. In the reverse Stroop task, in the leftmost column, a color name is printed in another color (e.g., “blue” is printed in green) and five other columns are painted in five different colors. The participants have to check the column whose color corresponds to the color name in the leftmost column. In the Stroop task, in the leftmost column, a color name is printed in another color (e.g., “blue” is printed in green) and five other columns contain color names. The participants have to check the column with the word corresponding to the name of the color in which the word in the leftmost column is printed (Supplemental Figure 1). During each task, the participants were instructed to complete as many tasks as possible in 1 min. Four tasks were performed in a fixed order, but the order of the task did not affect the performance of each task (Hakoda and Sasaki, 1990). We used the Word-Color and Color-Word tasks as simple processing speed measures and Stroop and reverse Stroop tasks as inhibition measures (Takeuchi et al., 2011d).
[H] The S-A creativity test (Society_for_Creative_Minds, 1969) is a creativity test measured by divergent thinking. A detailed discussion of the psychometric properties of this instrument and how it was developed is found in the technical manual of this test (Society_for_Creative_Minds, 1969). The test is used to evaluate creativity through divergent thinking (Society_for_Creative_Minds, 1969) and involves 3 types of tasks that require subjects to (1) generate unique ways of using typical objects; imagine desirable functions in ordinary objects; and imagine the consequences of “unimaginable things” happening. The S-A test scores the 4 dimensions of the creative process (fluency, originality, elaboration, and flexibility). We used the sum of the graded scores of these 4 dimensions in the analysis. In the grade score, each dimension of the test was scored from 0 to 10. The nature of the S-A creativity test is similar to that of the Torrance Test of Creativity Thinking (TTCT), which is internationally known more widely, in that it consists of three problems, which are similar to three problems in the TTCT (Torrance, 1966). In these problems the subjects are asked to (1) improve a product (list ways to change a certain product so that it will have more desirable characteristics), (2) find interesting and unusual uses for a certain object, and (3) list all the consequences should an improbable situation occur (Torrance, 1966). For more details including the psychometric properties of this test, sample answers to the questionnaire, and the manner in which they were scored, see our previous works (Takeuchi et al., 2010a,b).

Tests A–H were administered to all groups and were thus used in this study. Other tests were as follows:

[I] The arithmetic task in the Japanese version (Fujita et al., 2006) of the Wechsler Adult Intelligence Scale-Third Edition (WAIS-III) (Wechsler, 1997) is a complex working memory task using mental calculation.
[J] The digit symbol task in the Japanese version (Fujita et al., 2006) of WAIS-III (Wechsler, 1997) is a processing speed task.
[K] The letter mental rotation task (Takeuchi et al., 2011c) is a mental rotation task using Japanese letters. For details, see our previous study (Takeuchi et al., 2011c).
[L] The letter span task, a verbal working memory task. This test is conducted in a manner similar to the Digit span task (Wechsler, 1997), except that instead of digits, Japanese letters are used. This measure was taken to rule out the possibility that the expected improvement in this task following training resulted because participants became habituated to remembering numbers (Takeuchi et al., 2011a).
[M] Trail Making Test parts A and part measure processing speed and executive function (cognitive flexibility; Kortte et al., 2002), respectively.
[N] Cattell's Culture Fair Test (Cattell and Cattell, 1973) is a non-verbal reasoning test.
[O] A computerized digit span task is a verbal working memory task (for the detail of this task, see Takeuchi et al., 2011b).
[L] A computerized visuospatial working memory task measures visuospatial working memory capacity (Takeuchi et al., 2011d).
[M] The Tanaka B-type intelligence test (Tanaka et al., 2003). Type 3B, which is for 3rd-year junior high school and older examinees, is a non-verbal mass intelligence test that does not include story problems but figures, single numbers, and letters as stimuli. In all subtests, subjects had to complete as many problems as possible within a few minutes. For the detail of this task, see our previous work (Takeuchi et al., 2011d).
[N] A computerized auditory backward operation span is a complex verbal working memory span task. Pairs of single-digit numbers are presented auditorily. Subjects have to add each pair of numbers and remember the sequences of the first digit of answers of these additions. They have to reverse the remembered sequences when they answer.

We collected several questionnaires designed to assess the traits or states of the subjects, but these are not described in this study. These questionnaires were mostly self-reporting questionnaires including participants' behaviors in their daily lives and were mostly designed to assess the traits of subjects and not the effect in the 6-day intervention period. Besides self-reporting questionnaires, all neuropsychological assessments were performed by post-graduate and undergraduate students blinded to the group status of the participants.

### Image acquisition

All MRI data were acquired using a 3-T Philips Achieva scanner. Using a magnetization prepared rapid gradient echo sequence, high-resolution T1-weighted structural images (240 × 240 matrix; repetition time, 6.5 ms; echo time, 3 ms; field of view, 24 cm; slices, 162; slice thickness, 1.0 mm) were collected. All study subjects also participated in other studies or projects, and MRI scans not described here were performed together with scans described in the previous study.

### VBM pre-processing

To investigate brain structural changes that are associated with control groups, VBM was used. VBM is a method for the *in vivo* study of human brain structures and can detect training-induced brain structural changes (Draganski et al., 2004). The morphological data were preprocessed using Statistical Parametric Mapping software (SPM8; Wellcome Trust Centre for Neuroimaging, London, UK), implemented in Matlab (Mathworks Inc., Natick, MA, USA) with the help of VBM8 software (http://www.neuro.uni-jena.de/vbm/download/).

The actual procedures that we took are as follows:

1. Pre- and post-T1 weighted structural images are coregistered to SPM8's “T1” template image independently (without reslicement). By this procedure, 2 images are realigned well.
2. Coregistered pre- and post-T1-weighted structural images are resliced to the same voxel size and dimensions of our normalized 1-mm-voxel T1-weighted structural image. Reslicement was performed to the normalized 1-mm-voxel T1-weighted structural image instead of SPM8's “T1” template image (which has a 2-mm^3^ voxel size) so that we can take advantage of the original 1-mm voxel size in the following preprocessing.
3. The mean image of the resliced pre- and post-images that were created in procedure (2) was created.
4. The unresliced pre- and post-images [images before procedure (2)] were coregistered and resliced to the mean image that was created in procedure (3).
5. The mean image of the coregistered and resliced pre- and post-images that were created in procedure (4) was created. This mean image was created and used in the following procedure [instead of the mean image that was created in procedure (3)] to create the mean image from the pre- and post-images that were perfectly aligned.
6. Next, using the function of VBM8, intra-subject bias correction was performed. Here, the reference image is the mean image created in procedure (5), and using this image, we corrected the bias of the coregistered and resliced pre- and post-T1-weighted structural images created in procedure (4).
7. Next, using the “new segmentation option” in SPM8, the mean image of the pre- and post-image that was created in procedure (5), bias-corrected pre- and post-images that were created in procedure (6) were segmented. Here, we used the default option, but affine regularization was performed using the International Consortium for Brain Mapping (ICBM) template for East Asian brains. Further, we used the GM tissue probability map (TPM), which lowered the signal intensity of the region immediately outside the cerebral parenchyma, to prevent the dura matter from being classified as GM (for details, Takeuchi et al., 2015).
8. Next, we realigned segmented GM images of the bias-corrected pre- and post-images to the segmented GM image of the mean image of pre- and post-images.
9. We then proceeded to the diffeomorphic anatomical registration through the exponentiated lie algebra (DARTEL) registration process implemented in SPM8. In this process, we used DARTEL-imported images of the 5 TPMs of the mean image of the pre- and post-image created using the aforementioned new segmentation process. The template for DARTEL was created using a portion (60 subjects) of the entire subjects who participated in a particular study (Takeuchi et al., 2011d) so that the number of men and women was approximately equal. Next, using this existing template, DARTEL procedures were performed using all subjects' mean images of pre- and post-T1-weighted images and default parameter settings. The resulting images were then spatially normalized to the MNI space to obtain images with 2 × 2 × 2 mm^3^ voxels. Next, using the normalization parameters created from these DARTEL procedures and the procedure of normalization to the MNI space, all subjects' segmented GM images of bias corrected pre- and post-images were normalized to obtain images with 2 × 2 × 2 mm^3^ voxels.
10. Subsequently, pre- and post-normalized GM images were smoothed by convolving them with an isotropic Gaussian kernel of 12 mm full width at half maximum, which is a relatively large value for the reasons described later in the text.
11. Finally, the signal change in rGMD between the pre- and post-intervention images was computed at each voxel for each participant. In this computation, we included only voxels that exhibited rGMD values of >0.10 in both pre- and post-MRI scans. The resulting maps representing the rGMD change between the pre- and post-MRI scans were then subjected to group-level analyses. Further smoothing is not applied here. Note, in these kinds of longitudinal preprocessing, modulation is not deemed necessary by developers of VBM and as were the cases of longitudinal analyses of VBM2 and VBM8, the modulation (Ashburner and Friston, 2000) is not performed here (http://www.neuro.uni-jena.de/vbm/download/).

These procedures before smoothing followed the procedure described in the widely distributed manual of VBM8 excluding the following points.

- As the first step of the alignment of pre- and post-images, we did not use the realignment to the mean image option of SPM8. Instead, we used the coregistered pre- and post- image to the template independently and resliced them to the 1-mm^3^ voxel.
- For the next step, we also used the coregister option to align the original pre- and post-images to the mean image instead of SPM8's realignment to the mean image option. These are because of the points (b) and (c) which were described below in this subsection and to achieve an unbiased registration process.
- We did not use the segmentation and normalization function of VBM8 because of the apparent failure in VBM8's segmentation of our image and replaced it with the previously described segmentation and normalization function of SPM8 (Takeuchi et al., 2015).

In this study, we utilized the procedures adjusted based on the one described in the manual of VBM8's longitudinal preprocessing. However, because of the following facts, we needed to develop our own procedures.

- As described previously (Takeuchi et al., 2012a, 2013, 2014), the segmentation process of VBM8 did not work well with our images for unclear reasons. Therefore, we were not able to use VBM8. The possible reason why the newer versions of VBM could not process our structural images but could process other low-quality images in our laboratory was explained in our previous study (Takeuchi et al., 2012a). Basically, we assume that GM, white matter, and cerebrospinal fluid of images must have contrasts that are similar to the supposed T1-weighted structural images to be processed in the newer version of VBM. Recently, however, we successfully utilized SPM8's “new segmentation” procedure and DARTEL registration process to our T1-weighted structural images after minor modifications (Takeuchi et al., 2013).
- The coregistration of the raw images to the template images often prevents the failure of subsequent preprocessing. Thus, in longitudinal preprocessing, we preferred the coregistration and reslicement of pre- and post-images to normalized template images and space to halfway registration (realignment of pre- and post-images to the mean space of pre- and post-images; Thomas and Baker, 2012) as the first step of preprocessing.
- The realignment procedures of SPM8, which are described in VBM8's manual, are biased because the realignment is performed to “mean” images of pre- and post-images. This is because in SPM8, this “mean” image is created after the realignment of the second image to the first image, which means the first and second images are going through different procedures.

### Statistical analyses of behavioral data

Behavioral data were analyzed using SPSS 18.0 (SPSS Inc., Chicago, IL, USA). First, we used One-way ANOVAs to investigate the possible group differences in performance changes in these cognitive tests following the 6-day intervention period. Next, in behavioral analyses in which all control group data were combined, we used paired *t*-tests (one-tailed) to assess whether performance on selected cognitive tests changed. In all behavioral analyses in this study, results with a threshold of *P* < 0.05, corrected for the false discovery rate (FDR) using the graphically sharpened method (Benjamini et al., 2006), were considered statistically significant. The correction for multiple comparisons using this method was applied among results of the 8 aforementioned paired *t*-tests, which tested the significance of the pre- to post-changes in performance on the cognitive tests, among 8 ANOVAs that tested pre-existing group differences of cognitive performance. FDR is the error rate in a set of comparisons that are called significant, or in other words, the proportion of comparisons that are wrongly called significant. In other words, among the multiple tested results, 5% of the results determined to be significant through this method are not truly significant. In FDR testing, if there is truly no signal anywhere in the tested results, an FDR-controlling method has the same control as a family-wise error correction. FDR-based methods have been found to be more powerful and sensitive than other available approaches to multiple statistical testing (See Benjamini and Hochberg, 1995 for a full discussion; Genovese et al., 2002).

### Statistical analyses of whole brain imaging data

Imaging data analysis were performed using SPM8.

First, to investigate the possible group differences among 4 control groups in rGMD changes following the 6-day intervention period, we used whole-brain One-way ANCOVA. The maps representing the rGMD change between the pre- and post-MRI scans created in this study were subjected to group-level ANCOVA. In this analysis, sex, age, total intracranial volume, and whether the subjects completed the pre-cognitive tests before the pre-MRI scan on the first day were included as covariates.

Second, we also tested differences in rGMD changes between the participants for whom the scan was acquired before or after the first assessment test. In this analysis, sex, age, and total intracranial volume were included as covariates. For these whole-brain ANCOVAs, correction for multiple comparisons were performed using the voxel-level family wise error (cluster-level statistical tests cannot be used in ANCOVAs).

Third, in the whole-brain analysis, using a one-sample *t*-test, we investigated regions that showed increased or decreased rGMD following the 6-day intervention period. In this analysis, sex, age, total intracranial volume, and whether the subjects completed the pre- cognitive tests before the pre-MRI scan on the first day were included as covariates. One-sample *t*-test was chosen so that the analysis can include abovementioned covariates easily.

In all whole-brain imaging analyses, the statistical significance level was set at *P* < 0.05, corrected at the non-isotropic adjusted cluster level [family wise error (F.W.E)] (Hayasaka et al., 2004) with an underlying voxel level of *P* < 0.0025. In this non-isotropic cluster-size test of the random field theory, a relatively higher cluster-determining threshold combined with high smoothing values of more than 6 voxels was demonstrated to lead to appropriate conservativeness in real data (Silver et al., 2012). With high smoothing values, an uncorrected voxel-level threshold of *P* < 0.01 appears to lead to rather less conservative cluster level statistical values, whereas a threshold of *P* < 0.001 appears to lead to “slightly” conservative cluster level statistical values (Silver et al., 2012).

### Statistical analyses to test associations between rGMD changes and cognitive changes through non-whole brain analyses

Next, to investigate the association between observed rGMD changes in the control groups and improvements related to cognitive tests, we performed (non-whole brain) correlation analyses. We used SPSS 18.0 (SPSS, Inc., Chicago, IL, USA) for these analyses. For these analyses, we extracted the mean values of rGMD changes in the cluster that was found to be significant in the 1-sample *t*-test analysis.

Simple regression analyses were performed to assess the amount of pre- to post-test performance changes in cognitive tests used as outcome measures and the mean rGMD changes in the significant cluster. In these regression analyses, to test the hypothesis described in the Introduction, 1-tailed tests were used to test the positive correlation between improvements in performance of the cognitive tests and mean changes in rGMD in the significant clusters that were identified in the 1-sample *t*-test that was described previously. For this purpose, all of the cognitive tests used as outcome measures in all 4 control groups were selected. These tests were RAPM, the simple arithmetic task, the complex arithmetic task, the Word-Color task, the Reverse Stroop task, the Color-Word task, the Stroop task, and the S-A creativity test. Only these tests were chosen because a number of identified significant areas of brain structural changes multiplied by a number of tests will substantially increase the number of comparisons in the correlation analyses and thus the risk of false positives.

In these regression analyses, results with a threshold of *P* < 0.05, corrected for the FDR using the graphically sharpened method (Benjamini et al., 2006), were considered statistically significant. A correction for multiple comparisons using this method was applied to the results of the eight regression analyses that tested the association between brain structural changes and changes in cognitive performance.

Note (a) this regression analyses that tested whether there were no associations between structural change and changes of the cognitive tests' performance, are orthogonal to (b) the one-sample *t*-test that tested the rGMD change was not zero, and therefore the present analyses are not relevant to the controversy regarding “double dipping” issues raised by Vul et al. (2009). This is because the first paired *t*-tests merely tested the average change in rGMV in the group after the intervention period, and this analysis did not involve any variables related to cognitive test performance in the statistical model or group comparisons. Furthermore, the two models were not related (one was a test regarding whether the average was greater than 0, and the other was a test of the associations between variables, regardless of whether the average of the variables was greater than, smaller than, or equal to 0). To more specifically explain this concept in a qualitative manner, in whole-brain analyses involving a one-sample *t*-test, overfitting occurs in areas of significant signal increases (Vul et al., 2009); however, this overfitting occurs to randomly increase all subjects' signals (in terms of individual differences among this group). There is only one group in both the one-sample *t*-test and regression analyses, this overfitting cannot associate any individual differences among this group in a biased manner. As these analyses do not involve group comparisons, for these signal changes in rGMV to be related to individual increases in cognitive performance, real associations between neural changes and cognitive changes would be required.

## Results

### Behavioral data

First, we used One-way ANCOVAs to investigate the possible group differences in performance changes in the abovementioned cognitive tests after the 6-day intervention. No significant group differences were observed (Table 2).

**Table 2:** **Pre- and post-test performance and performance changes in psychological measures**.

	**Non-adaptive low-level active control group (*N* = 17)**	**Passive control group 1 (*N* = 18)**	**Passive control group 2 (*N* = 19)**	**Passive control group 3 (*N* = 28)**	***P*-values^†^ and effect size**	***P*-values^‡^**
RAPM^*^ (items^**^)	29.12 ± 3.45 (22–34)	27.83 ± 2.95 (22–32)	28.26 ± 3.96 (21–35)	28.57 ± 3.34 (21–33)	6.68 × 10^−14^(0.843)	0.480
	32 ± 3.12 (26–36)	31.39 ± 2.48 (26–36)	30.32 ± 4.01 (21–36)	31.57 ± 3.36 (21–36)		
	2.88 ± 2.91 (−2–12)	3.56 ± 1.95 (0–7)	2.05 ± 2.09 (−3–6)	3 ± 3.68 (−2–12)		
Simple arithmetic (items)	32.97 ± 5.62 (24.5–47)	34.42 ± 5.15 (25.5–47.5)	33.34 ± 4.68 (25–42.5)	29.73 ± 4.71 (20.5–40.5)	6.07 × 10^−6^ (0.351)	0.189
	35.38 ± 3.45 (29–42)	34.64 ± 5.14 (26.5–45.5)	35.66 ± 5.04 (22.5–45.5)	31.88 ± 4.92 (23.5–45)		
	2.41 ± 5.29 (−10–10.5)	0.22 ± 3.02 (−4.5–8)	2.32 ± 2.35 (−2.5–6)	2.14 ± 2.70 (−5.5–7.5)		
Complex arithmetic (items)	7.06 ± 2.17 (2–10.5)	8.06 ± 3.06 (4.5–16)	7.16 ± 1.88 (3.5–10)	6.95 ± 4.36 (2–26)	8.67 × 10^−4^ (0.189)	0.850
	7.38 ± 2.69 (1–12.5)	8.92 ± 3.75 (4.5–17)	7.92 ± 1.97 (5–11.5)	7.64 ± 5.12 (2–31)		
	0.32 ± 2.77 (−6.5–4)	0.86 ± 1.72 (−2–4)	0.76 ± 1.19 (−1–4)	0.70 ± 1.59 (−2.5– 5)		
Word-Color task (items)	73.06 ± 6.62 (62–89)	70.61 ± 8.58 (57–87)	73.79 ± 5.43 (67–85)	68.75 ± 8.13 (53–90)	3.04 × 10^−16^ (0.746)	0.977
	79.35 ± 6.07 (68–87)	76.22 ± 10.99 (50–100)	80.16 ± 5.23 (75–93)	74.93 ± 9.43 (59–100)		
	6.29 ± 3.82 (−2–12)	5.61 ± 8.24 (−20–16)	6.37 ± 4.36 (−2–14)	6.18 ± 4.69 (−4– 14)		
Reverse Stroop task (items)	62 ± 8.72 (48–79)	57.28 ± 8.32 (38–75)	62.21 ± 6 (52–74)	56.54 ± 10.3 (31–79)	3.06 × 10^−8^ (0.457)	0.979
	66.59 ± 6.6 (58–83)	59.67 ± 8.64 (46–77)	66.89 ± 4.93 (58–75)	60.68 ± 8.96 (45–76)		
	4.59 ± 4.67 (−5–17)	2.39 ± 7.32 (−13–14)	4.68 ± 3.74 (−3–12)	4.14 ± 6.78 (−12– 22)		
Color-Word task (items)	52.94 ± 7.18 (43–69)	50.39 ± 4.92 (43–58)	53.58 ± 5.32 (43–62)	51.46 ± 7.2 (36–65)	1.25 × 10^−8^ (0.436)	0.901
	55.88 ± 6.52 (45–66)	53.28 ± 5.65 (45–64)	56.11 ± 6.73 (44–66)	54.54 ± 7.24 (39–69)		
	2.94 ± 5.54 (−6–15)	2.89 ± 4.48 (−7–12)	2.53 ± 3.14 (−3–9)	3.07 ± 3.63 (−3– 11)		
Stroop task (items)	49 ± 6.68 (33–61)	45.39 ± 6.77 (35–60)	48.68 ± 5.7 (40–62)	46.14 ± 7.01 (31–63)	3.48 × 10^−8^ (0.464)	0.258
	51.18 ± 6 (41–62)	47.28 ± 6.17 (34–62)	51.95 ± 6.26 (38–61)	50.61 ± 7.29 (35–68)		
	2.18 ± 4.15 (−3–12)	1.89 ± 4.45 (−9–8)	3.26 ± 5.66 (−7–15)	4.46 ± 4.3 (−4–13)		
S-A creativity task (total grade scores^***^)	26.88 ± 6.98 (17–39)	23.61 ± 5.32 (14–31)	26.21 ± 6.17 (11–36)	23.29 ± 5.34 (12–34)	0.0170 (0.187)	0.727
	27.29 ± 5.89 (16–36)	24.72 ± 5.57 (16–33)	26.84 ± 5.74 (12–37)	25.18 ± 5.99 (10–36)		
	0.41 ± 4.75 (−8–8)	1.11 ± 3.68 (−6–9)	0.63 ± 5.82 (−11–11)	1.89 ± 4.21 (−6–11)		

*Data are given as the mean ± SD (range, minimum–maximum; the upper line, pre-test data; the middle line, post-test data; the lower line, Post—Pre change)*.

* *Raven's Advanced Progressive Matrices (Raven, 1998)*.

** *Number of items solved in the test time*.

*** *Sum of the graded scores of these 4 dimensions in the analysis. In the grade score, each dimension (fluency, originality, elaboration, flexibility) of the test was scored from 0 to 10 (See Methods for more details)*.

† *Paired t-tests (one-tailed) in which data from the 4 groups were combined. P values of the false discovery rate (FDR, corrected) using the 2-stage sharpened method are null because all of the uncorrected P-values are < 0.05 (meaning due to the nature of this FDR testing, when all of the tested analyses exhibit an uncorrected P-value of < 0.05, in FDR, the corrected P-values should all be < 0.05, and this FDR testing does not return the statistical values)*.

‡ *One-way ANOVAs to test the possibility of group differences in test-retest differences in psychological measures. P-values of FDR (corrected) using the 2-stage sharpened method are close to 1 because all of the uncorrected P-values are well above 0.10 (meaning due to the nature of this FDR testing, when all the tested analyses exhibit an uncorrected P-value well above 0.10, in this FDR testing, corrected P-values should all be >0.10, and this FDR testing does not return the statistical values)*.

For behavioral analyses in which all control groups' data were combined, we used paired *t*-tests to assess whether performance in cognitive tests, which were conducted before and after a 6-day intervention in the control groups of previous studies, had changed. The performance in all the cognitive tests that were investigated in this study significantly improved after a 6-day intervention period (Table 2). Note that for the S-A creativity task, there are two versions that can be administered as pre- and post- tests, and the problems comprising the tasks used in the pre-test (A version) and the tasks used in the post-test (C version) are different (for details, see Society_for_Creative_Minds, 1969; Takeuchi et al., 2010b).

### Increase in rGMD after the 6-day intervention period in control groups

First, we performed One-way ANCOVAs of whole-brain data to investigate possible group differences in rGMD changes among the four control groups after the 6-day intervention period. No significant group differences in rGMD changes were observed among the four groups.

Next, we performed One-way ANCOVAs of whole-brain data to test the differences in rGMD changes between participants for whom scans were acquired before or after the first assessment test. No significant group differences in rGMD changes were observed. Mostly, subjects underwent MRI and the tests in a fixed order in the pre- and post-assessment periods, and thus, the specific effect of the order of the second scan could not be evaluated.

**Figure 1:**
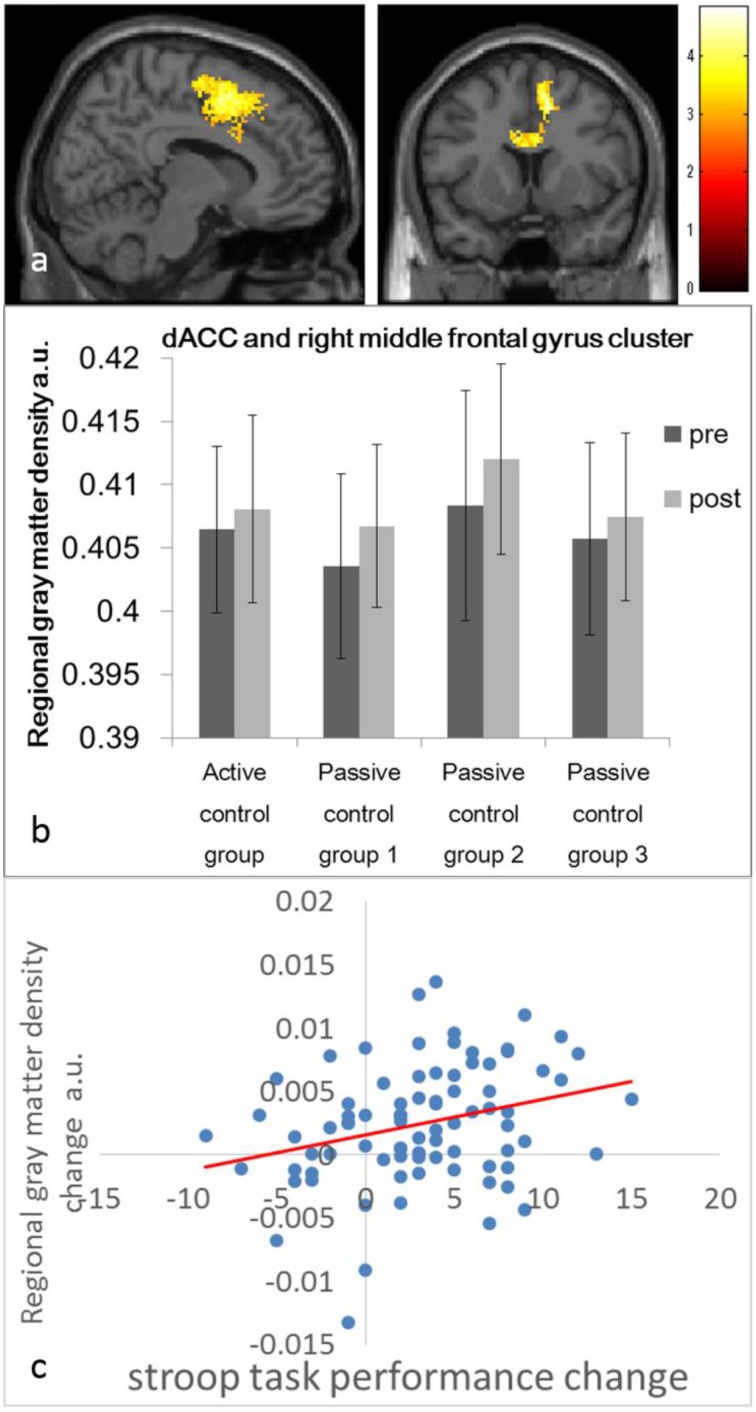
**Regional gray matter density (rGMD) increases and their association with improvement in the performance of cognitive tests in the control groups. (A)** rGMD increase in the control groups. Results are shown with *P* < 0.05 corrected for multiple comparisons at the non-isotropic adjusted cluster level with an underlying voxel level of *P* < 0.0025 (uncorrected). **(B)** The histogram presents the mean rGMD values of the significant cluster that spread around the bilateral dorsal anterior cingulate cortices and the right superior frontal gyrus before and after the intervention period for each control group. Error bars represent standard deviations. **(C)** A scatter plot between the improvement of Stroop test performance and the mean rGMD value in the significant cluster in **(A)** is presented for visualization only.

In whole-brain analyses, in which data from the four control groups were combined, we used one-sample *t*-test to assess whether rGMD changed after the 6-day intervention period in control groups. A significant increase in rGMD was observed in an anatomical cluster that extended to the bilateral dACC and right superior frontal gyrus (*x, y, z* = 12, 4, 50; *t* = 4.83; *P* < 0.001, corrected for multiple comparisons at the cluster level, with a cluster-determining threshold of *P* < 0.0025, uncorrected; Figures 1A,B).

Furthermore, rGMD [>100 voxels (800 mm^3^)] tended to increase in some of the regions, in accordance with our hypothesis. These regions included an anatomical cluster in the left inferior parietal lobule (*x, y, z* = −24, −48, 42; *t* = 4.56; 119 voxels, *P* < 0.0025, uncorrected), an anatomical cluster in the right superior frontal gyrus (*x, y, z* = 22, 54, 44; *t* = 4.04; 106 voxels, *P* < 0.0025, uncorrected), and an anatomical cluster that mainly spread in and around the right inferior parietal lobule (*x, y, z* = 48, −22, 36; *t* = 3.97; 185 voxels, *P* < 0.0025, uncorrected). Please note that this is a report of the findings regarding insignificant tendencies in areas of our a priori hypothesis; therefore, we did not draw any conclusions from the results of this study. No areas exhibited a significantly reduced rGMD.

Although this analysis suggested that rGMD increased in both the active control and passive control groups, this did not contradict the results of analyses that compared whether group differences in rGMD changes among control groups existed and that identified no group differences. This analysis suggested that rGMD increased over the baseline in the control groups, whereas the group difference analyses revealed no group differences in rGMD changes from the baseline.

### Association between increases in rGMD and performance changes in cognitive tests

Regarding the cluster that exhibited a significant increase in rGMD after the 6-day period, we performed simple linear regression analyses of mean rGMD changes in this cluster and performance changes in cognitive tests.

The mean rGMD change in this cluster exhibited a significant correlation with performance changes in the Stroop task (*P* < 0.05, corrected for FDR) and a trend toward a correlation with performance changes in the S-A creativity test (*P* = 0.0561, uncorrected). For all results and statistical values, refer to Table 3. No significant negative correlations were observed.

**Table 3:** **Statistical values of simple regression analyses between mean regional gray matter (GM) changes in the cluster that mainly involve the dorsal anterior cingulate cortex and right superior frontal gyrus, which exhibits significant pre- to post-test increases in regional GM and test-retest differences in cognitive tests**.

	***P*-value (uncorrected)**	***P*-value (corrected for false discovery rate)**	***T*-value**	***r*-value**
RAPM	0.886	0.814	−1.213	−0.134
Simple arithmetic	0.715	0.751	−0.571	−0.0637
Complex arithmetic	0.335	0.666	0.427	0.0477
Word-Color task	0.675	0.751	−0.455	0.0508
Reverse Stroop task	0.572	0.751	−0.181	−0.0202
Color-Word task	0.363	0.666	0.353	0.0394
Stroop task	0.00419	0.0308	2.704	0.289
S-A creativity test	0.0561	0.206	1.606	0.177

*RAPM, Raven's Advanced Progressive Matrices*.

## Discussion

The present study revealed that rGMD increases occur in control groups after a 1-week intervention in the young healthy adults. Consistent with our hypothesis, a statistically significant increase in rGMD was observed in an anatomical cluster that that extended to the bilateral dACC and right superior frontal gyrus. Moreover, across all these affected regions, increases in rGMD were associated with increases in performance on Stroop task. Thus, our results indicate that the observed rGMD changes are associated with improvements related to Stroop task.

We conclude that even in the groups of the typical “control group” in intervention studies including those of the passive one, experimental or non-experimental factors can result in an increase in the regional GM structure and form the association between such neural changes and improvements related to these cognitive tests. These results suggest caution toward the experimental study designs without control groups. One possible mechanism to cause such a change is of course, exposure to cognitive tests as we presumed in Introduction, however these cannot be concluded from the study. This is because there can be a time-related change of brain structure and since there are no groups that did not go through the exposure to cognitive tests in this study.

The present findings do not cast doubt on previous findings that used the control groups to investigate the effects of intervention on brain structures (Takeuchi et al., 2011c). They are just amount to the fact that like the repeated measurement of psychological tests itself improve the performance of those tests, just going through the experiment (possibly including psychological assessments) can affect brain structures. So as long as control groups were used, it does not matter like in the case of investigation of intervention effects on psychological measures. But the present findings may cast doubt on future intervention studies without control groups as well as those studies that tried to investigate the effects of intervention through the associations between brain structural changes and changes of performance of psychological outcome measures without using control groups. This is because without intervention itself, changes of brain structure and changes of performance of psychological measures can associate.

The changed brain areas are involved in a wide range of cognitive operations. Thus, the pre- to post-test performance changes in cognitive measures, which have been believed to simply reflect learning effects, may be partly affected by an increase in neural functions caused by exposure to cognitive tests.

We speculated that a possible mechanism underlying the observed structural changes is the usage-dependent genesis of synapses. Very rapid experience-dependent structural changes (hours to days after experience) occur continuously at the level of synapses (Feldman, 2009). Animal studies also showed that experience-dependent synaptogenesis can occur well within the experiment period (Trachtenberg et al., 2002; Alvarez and Sabatini, 2007), and together with synaptic elimination, underlie daily experience-dependent neural plasticity (Trachtenberg et al., 2002; Alvarez and Sabatini, 2007). Potential regional GM structural correlates include the synaptic bulk level (Draganski et al., 2004; May and Gaser, 2006). Thus, increased bulk of synapses may cause the regional GM increases observed in this study.

rGMD may dynamically change based on daily experiences. If just a 1-day exposure to cognitive tests is sufficient to cause rGMD changes, these structural changes may occur following a wide range of cognitive activities. Experience-dependent synaptic and spine changes occur dynamically based on daily experiences and are mostly transient (Trachtenberg et al., 2002; Alvarez and Sabatini, 2007). Thus, considering these facts, if the observed structural alterations are based on synaptic and spine changes, the observed structural changes may be transient, implying that rGMD are changing dynamically based on daily experiences and that these changes occur in a span of days or hours similar to spine and synaptic changes (Trachtenberg et al., 2002; Alvarez and Sabatini, 2007).

On the other hand, unlike exposure to cognitive measures, up to 20 h of concentrated low level non-adaptive training did not have any impact on regional GM structure in our previous study (Takeuchi et al., 2011c). This might suggest that certain conditions have to be met to elicit structural brain changes. Non-adaptive low-level training does not lead to improvements in performance on untrained cognitive tasks (Klingberg et al., 2002); our previous study results were consistent with this finding (Takeuchi et al., 2011c). In our previous study, the low level non-adaptive training group underwent 20-h non-adaptive low-level working memory training, contacted experimenters more frequently than the passive control group, had confidence in the effects of intervention as much as the group undergoing actual training. However, despite all these factors, low level non-adaptive training did not lead to greater structural brain changes or performance changes in untrained cognitive tasks compared with passive control (Takeuchi et al., 2011c). This was tested in this previous study. The present study used different preprocessing methods, but the additional analysis using the rGMD images created in this study led to the same conclusion. The passive control and low level non-adaptive training groups were exposed to the same cognitive outcome measures. Thus, this type of cognitive training may not be sufficient to cause structural brain changes, and certain conditions (in this case, exposure to challenges greater than the capacity of an individual's performance) may have to be met to elicit the structural brain changes.

GM structural changes in dACC and the right superior frontal gyrus may contribute to performance changes by facilitating the function of these regions. rGMD changes in the anatomical cluster involving dACC and the right DLPFC were significantly correlated with performance changes in the Stroop task and displayed a tendency toward association with performance changes in the S-A creativity test. These two regions are consistently activated during Stroop tasks (Laird et al., 2005) and divergent thinking tasks (Dietrich and Kanso, 2010). dACC is involved in performance monitoring (Carter and Van Veen, 2007), and the right DLPFC is involved in the manipulation or mental operation of objects retained in the mind of an individual (Hagler and Sereno, 2006), including the operation and regulation of attention as well as generation of novel associations (Kiefer et al., 1998; Seger et al., 2000), which are apparently important for tasks such as the Stroop task and/or divergent thinking tasks. Perhaps through these functions, identified brain areas may be associated with cognitive performance.

In this study, among the regions of the *a priori* hypothesis, only the anatomical cluster around dACC and the right DLPFC displayed a significant rGMD increase, and only the performance change in the Stroop task was significantly correlated with the mean rGMD change in this cluster (a trend toward correlation was observed for the S-A creativity task). However, as shown in Results, the tendency of rGMD was observed in other areas of the regions of the *a priori* hypothesis, namely the bilateral inferior parietal lobules and another area in the right DLPFC. Thus, other areas in the fronto-parietal network may exhibit an increase in rGMD following exposure to cognitive tests. These suprathreshold areas of rGMD increase might be associated with performance improvement of other cognitive tasks. Further, the characteristics common to the Stroop task and the S-A creativity test may be that both of these tests are (a) speeded tasks and (b) involve cognitive operations that subjects usually do not encounter (such as Stroop rules and divergent thinking). Exposure to experiences involving these other processes may also affect brain structures. Alternatively, because dACC is activated by a wide range of externally directed attention-demanding cognitive tasks (Fox et al., 2005), it is possible that exposure to each cognitive task employed in the study impacted this structure slightly, but altered function of this area was only robustly associated with Stroop performance, as this area plays a specifically crucial role in this task as shown by meta-analyses of functional activation studies (Laird et al., 2005) as well as our structural study (Takeuchi et al., 2012b). Other factors such as statistical deviations and how reliably the test can estimate a person's cognitive abilities without ceiling effects might affect the differences in correlations between performance changes and structural brain changes.

Related to this point, whether the observed structural changes reflect generalized transfer effects is an interesting question, as only learning in cognitive measures that are likely to cause far transfer effects seems to be related to the observed structural changes. However, since structural changes can occur following cognitive intervention which is not known to cause generalized far transfer effects (Ilg et al., 2008), this question cannot be answered from the results of this study. Future well designed psychological tests should answer this question.

This study has at least 1 limitation. Here all groups were exposed to cognitive test outcome measure, and we showed the effects of exposure to these tests on rGMD by demonstrating an association between rGMD changes and performance changes in these tests. This method is widely used and is one of the standard methods used in imaging studies of cognitive intervention (Olesen et al., 2004; McNab and Klingberg, 2007; Takeuchi et al., 2010d). However, it is possible that experimental factors other than exposure to cognitive tests (such as exposure to MRI) affected rGMD as well as the cognitive test results and are responsible for the observed associations, although we are not aware of any such theoretically possible experimental factors. Future studies should investigate the extent of rGMD plasticity.

Finally, the identified areas are more extensive and involved more higher order cognitive areas than the areas identified for the effects of training of simple processing speed tasks (the perisylvian area and motor and visual areas; Takeuchi et al., 2011d; Takeuchi and Kawashima, 2012), such as the right superior frontal gyrus and the dACC. This might be due to the involvement of complex speeded cognitive tasks such as the Stroop task and a divergent thinking task in the outcome measures. Furthermore, related to this point, observed structural changes may be just caused by the “exposure to these speeded cognitive tasks” and contrary to our discussions, “exposure to cognitive tests” *per se*, may not cause the structural change. But these are speculative and since a lot of tests are administered and much of the improvements of performance as well as structural changes of different regions seem to be correlated with each other more or less and disentangling the associations among them in a definitive way is difficult in this present study. Training protocols for cognitive inhibitions tasks and divergent thinking tasks are known (Thorell et al., 2009). Thus, future studies can investigate the effects of these cognitive trainings on neural systems.

## Author contributions

HT, YT, and RK designed the study. HT, YS, AS, TN, RN, and AF collected the data. HT analyzed the data and prepared the manuscript.

## Compliance with ethical standards

As per the Declaration of Helsinki (1991), written informed consent was obtained from each subject and in case subjects are minor, his/her parent prior to MR scanning. The Ethics Committee of Tohoku University approved the study.

### Conflict of interest statement

The authors declare that the research was conducted in the absence of any commercial or financial relationships that could be construed as a potential conflict of interest.
