# Supplementary material for: The Associations between Regional Gray Matter Structural Changes and Changes of Cognitive Performance in Control Groups of Intervention Studies
Source: Front Hum Neurosci. 2015 Dec 21;9:681. doi: 10.3389/fnhum.2015.00681 (PMC4685061; doi:10.3389/fnhum.2015.00681)

Supplemental online material.

Supplemental Figure legend

Supplemental Fig. 1. Schema of tasks used in this study. There were two control tasks (a word-color task and a color-word task), a reverse Stroop task, and a Stroop task. This figure is reproduced from our previous publication [1].

References

1. Takeuchi H, Taki Y, Sassa Y, Hashizume H, Sekiguchi A, et al. (2012) Regional gray and white matter volume associated with Stroop interference: Evidence from voxel-based morphometry. Neuroimage 59: 2899-2907.

Supplemental Fig. 1.

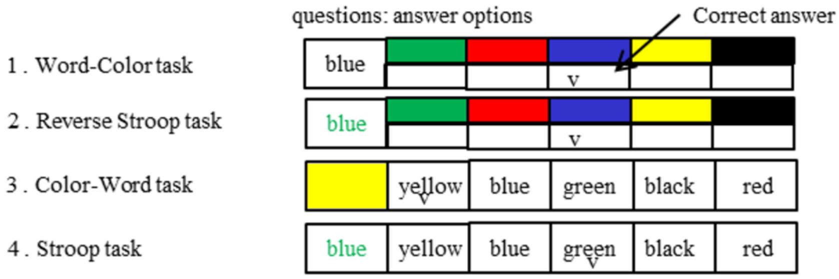

Supplement: Supplementary file 1 [file Image1.PDF]
